# Lower Central Fat Increase Risk of One-Year Muscle Mass Loss in Menopausal Women

**DOI:** 10.1155/2020/4650318

**Published:** 2020-09-02

**Authors:** Ying-Chou Chen, Wei-Che Lin, Tien-Tsai Cheng, Jia-Feng Chen, Shan-Fu Yu, Chung-Yuan Hsu

**Affiliations:** ^1^Department of Rheumatology, Kaohsiung Chang Gung Memorial Hospital, Chang Gung University College of Medicine, Kaohsiung 833, Taiwan; ^2^Department of Radiology, Kaohsiung Chang Gung Memorial Hospital, Chang Gung University College of Medicine, Kaohsiung 833, Taiwan

## Abstract

**Background:**

Hormonal changes had been found in menopausal women. Muscle and bone mass decline after menopause and with aging, increasing the risk for sarcopenia and osteoporosis in later life. Only a few studies suggest that menopausal hormonal changes have an effect on the decline in muscle mass.

**Objectives:**

This study aimed at evaluating the risk of muscle mass loss in menopausal women.

**Materials and Methods:**

Menopausal women from routine physical health examination were eligible for this study. Muscle mass was determined using dual-energy X-ray absorptiometry at baseline and 1 year later. All of the patients underwent the assessments for liver and kidney function, diabetes, and hypertension, and associated comorbidities were recorded.

**Results:**

A total of 172 patients were enrolled. 70 patients had muscle loss at 1 year, and the other 102 did not had loss. The mean age was 70.26 ± 9.93 years at the muscle loss group, while 69.25 ± 10.50 at the nonprogress group (*p* = 0.531). The mean body mass index was 22.96 ± 1.91 kg/m^2^ at the muscle loss group, while 23.33 ± 3.71 kg/m^2^ at the nonprogress group (*p* = 0.433). The baseline trunk limb fat mass ratio was 1.01 ± 0.20 in the muscle loss group and 1.12 ± 0.26 in the no muscle loss (*p* = 0.004). Using muscle mass loss as the outcome, logistical regression analysis showed that a baseline trunk limb mass ratio could predict muscle loss, and a higher baseline trunk limb mass ratio was associated with less muscle loss, while a lower trunk limb mass ratio was associated with increased muscle mass loss (*p* = 0.01).

**Conclusion:**

This is the first study to investigate the risk of muscle mass loss in menopausal women. Menopausal women with higher central fat had less muscle mass loss, while lower central fat was a risk factor for muscle mass loss. Chronic kidney disease was also a risk factor for muscle mass loss in menopausal women in this study.

## 1. Introduction

Hormonal changes such as an increase in serum follicle-stimulating hormone (FSH) and decrease in estradiol occur in menopausal women due to ovarian aging and the consequent menopausal transition [1, 2]. These hormonal changes start 5 years before and continue for years after the final menstrual period [3]. Therefore, studies on the effect of the menopause on women's health and well-being during aging are important. Muscle and bone mass decline after menopause and with aging, increasing the risk of sarcopenia and osteoporosis in later life. The hormonal changes in menopause are known to lead to bone loss through increased bone turnover with a net deficit in the bone formation relative to bone resorption [4, 5]. Several studies have also suggested that menopausal hormonal changes have an effect on the decline in muscle mass among middle-aged women [6, 7].

The abnormal body composition has been reported in individuals with sarcopenia [8, 9], and this has been associated with greater disability and poor long-term outcomes [10] and an increased risk of falls, hip fractures, decreased mobility, and reduced quality of life [11, 12]. However, few studies have examined serial changes in the body composition in menopausal women. Therefore, the aim of this study was to examine changes in the body composition at baseline and after 1 year to identify the factors influencing the body composition changes in menopausal women.

## 2. Materials and Methods

### 2.1. Study Population

This prospective survey was conducted at the Kaohsiung Chang Gung Memorial Hospital. Menopausal women from routine physical health examination between January 2017 and September 2018 were eligible for this study. Because we intended to examine the body composition changes, we excluded patients who had previously had a stroke and those with systemic infections.

### 2.2. Outcome Measures

We measured body weight, body mass index, muscle mass, and trunk limb fat mass ratio at baseline and 1 year later. Appendicular muscle mass and trunk limb fat mass ratio were measured using dual energy X-ray absorptiometry. All patients underwent the assessments for liver and kidney function, diabetes, and hypertension, and associated comorbidities were recorded. We defined those with a decrease in the appendicular muscle mass from baseline to 1 year as the muscle loss group, and those with no decrease as the no muscle loss group.

This study was approved by the Ethics Committee of the Kaohsiung Chang Gung Memorial Hospital, and all participants provided written informed consent. The study was conducted according to the tenets of the Declaration of Helsinki.

### 2.3. Statistical Analysis

Statistical analysis was performed using SPSS software, version 21.0 (SPSS, Chicago, IL, USA). Patient characteristics were reported as simple descriptive statistics (mean ± SD). Comparisons between independent means were analyzed using the *t*-test. Relationships between categorical variables were evaluated using the chi-square test. Logistic regression analysis was used to adjust for the potential confounding factors. A *p* value <0.05 was considered to be statistically significant.

## 3. Results

A total of 172 patients were enrolled, including 70 who had muscle loss at 1 year and 102 who did not. The mean age was 70.26 ± 9.93 years in the muscle loss group and 69.25 ± 10.50 in the no muscle loss group (*p* = 0.531). The mean body mass index was 22.96 ± 1.91 kg/m^2^ in the muscle loss group and 23.33 ± 3.71 kg/m^2^ in the no muscle loss group (*p* = 0.433). The baseline trunk limb fat mass ratio was 1.01 ± 0.20 in the muscle loss group and 1.12 ± 0.26 in the no muscle loss (*p* = 0.004). The muscle loss group had higher rates of diabetes (*p* = 0.016) and chronic kidney disease (CKD) (*p* = 0.012) than the no muscle loss group. The characteristics of the patients are shown in Table 1. Using muscle mass loss as the outcome, logistical regression analysis showed that a baseline trunk limb mass ratio could predict muscle loss, and a higher baseline trunk limb mass ratio was associated with less muscle loss, while a lower trunk limb mass ratio was associated with increased muscle mass loss (*p* = 0.01). Chronic kidney disease was associated with an increase in muscle loss (*p* = 0.041) (Table 2). In subanalysis, we found 6/8 (75%) of those with menopause within 5 years had muscle mass loss, while only 64/164 (39%) had muscle mass loss (*p* = 0.043).

## 4. Discussion

Hormonal changes can influence bone and muscle mass in menopause women [4, 5], and a change in the body composition has been associated with greater risks of poor health-related outcomes and disability [13]. Excess body fat has been associated with a decrease in muscle mass and strength with aging [14], leading to an increased risk of disability [15], institutionalization [16], and mortality [17]. However, these findings are in contrast to ours.

Adipocyte lipolysis has been reported to lead to muscle loss and the leakiness of the respiratory electron-transport chain and also to confer high energy expenditure. Unlike ATP synthase, uncoupling protein 1, which is only expressed in brown adipocytes, has been shown to speed up respiration and convert electrochemical energy into heat production [18]. Moreover, other mitochondria abnormities such as atrophy of oxidative capacity [19] may be involved in muscle weight loss. In a postgastrectomy study, an increase in cytokines was shown to enhance catabolism, leading to a sudden reduction in lean mass. Moreover, body weight was still reduced at least 1 month after surgery due to a reduction in fat mass, which was attributed to a reduced dietary intake [20]. In menopausal woman, dietary factors should also be considered to be an important factor leading to fat loss and muscle loss.

Excess adiposity has been reported to play a role in positive energy balance and decreases in all major components of the total energy expenditure [21] and physical activity [22]. This can then affect the development of sarcopenia, which is aggravated by the increased skeletal muscle fatty infiltration [23], reduced protein intake [24], impaired muscle energetics [25], the increased expression of myostatin [26], altered skeletal muscle substrate metabolism [27], and increased expression of myostatin [26]. However, the exact mechanism in menopausal women is still unclear. In the present study, an increased trunk limb fat mass ratio, meaning central fat, may have influenced the loss of muscle mass. In contrast to decreases in all major components of the total energy expenditure, few studies have reported on how to prevent muscle mass loss in menopausal women. Consequently, a multifaceted approach to the management of menopausal women remains the most promising in terms of reducing the associated health care burden from both a personal and public health perspective.

The key finding of this study is that a higher trunk limb fat mass ratio was associated with a lower risk of muscle mass loss. This may be because adipose fat can protect muscle loss, which has not been reported in other studies. We hypothesize that as female hormones are stored in fat tissue, more fat will result in less muscle loss and less osteoporosis. This is supported by a previous research in which increases in all adiposity indices were significantly associated with higher estimated bone mineral density and lower risk of osteoporosis [28]. In addition, another study reported that obesity may have a protective effect, because a higher body weight prevented the loss of muscle mass [29].

We also found that CKD was a risk factor for an increase in muscle mass loss. CKD has been associated with increased frailty and weakness, slow gait, exhaustion, low tolerance for physical activity, and unintentional weight loss [30], and also with muscle wasting, morbidity, and mortality [31]. Worsening the CKD stage and longer dialysis vintage have also been associated with a progressive loss of leg skeletal muscle mass [32]. It is found that chronic kidney disease, and especially dialysis and related lifestyle modification, can greatly impact weight and muscle mass loss. By different types of exercise program, it could be implemented in these patients to improve muscle mass loss [33–35].

The subanalysis of this study showed menopause with 5 years which had more muscle mass progress, so it could explain more about how to prevent the mentioned muscle loss and give directions to further studies that would investigate the interventions to prevent the process of muscle mass loss.

The limitations of this study include the single-center nature, the lack of data on dietary intake and long-term muscle progress, and the small sample size. The strengths of the study include that body composition was analyzed using dual energy X-ray absorptiometry, which is accurate for measuring the human body composition. Further studies are warranted to address the limitations of this study and verify our findings.

In conclusion, this is the first study to investigate the risk of muscle mass loss in menopausal women. Menopausal women with higher central fat had less muscle mass loss, while lower central fat was a risk factor for muscle mass loss. CKD increased the risk of muscle loss, and this is also an important factor in menopause. Clinicians should monitor muscle mass in menopausal women with lower central fat, because these women are prone to muscle loss.

## Figures and Tables

**Table 1:** The characteristics of the study patients.

Variables	Muscle mass loss (*n* = 70)	No muscle mass loss (*n* = 102)	*P* value
Age (years), mean ± SD	70.26 ± 9.93	69.25 ± 10.50	0.531
Body mass index (kg/m^2^), mean ± SD	22.96 ± 1.91	23.33 ± 3.71	0.433
Baseline trunk limb fat mass ratio	1.01 ± 0.20	1.12 ± 0.26	0.004
Smoking (*n*, %)	2 (2.9)	2 (2.0)	0.538
Alcohol consumption (*n*, %)	2 (2.9)	4 (3.9)	0.53
Diabetes mellitus (*n*, %)	18 (25.7)	12 (11.8)	0.016
Hypertension (*n*, %)	22 (31.4)	30 (29.4)	0.453
Cardiovascular disease (*n*, %)	10 (14.3)	10 (9.8)	0.253
Chronic liver disease (*n*, %)	6 (8.6)	12 (11.8)	0.342
Neurological disease (*n*, %)	2 (2.9)	6 (5.9)	0.296
Chronic kidney disease (*n*, %)	8 (11.4)	2 (2.0)	0.012

**Table 2:** Factors influencing muscle mass loss.

Variables	Regression coefficient	SE	Wald	*p* value	OR (95% CI)
Trunk/limb fat mass ratio	-3.14	0.86	13.46	0.001	0.043 ± (0.008 − 0.231)
Diabetes mellitus	0.75	0.50	2.23	0.135	2.126 ± (0.790 − 5.718)
Chronic kidney disease	2.12	1.03	4.19	0.041	8.300 ± (1.094 − 62.985)

Abbreviations: OR: odds ratio; SE: standard error.

## Data Availability

All the dataswere availabel as reguested.
